# Population Bottlenecks as a Potential Major Shaping Force of Human Genome Architecture

**DOI:** 10.1371/journal.pgen.0030119

**Published:** 2007-07-20

**Authors:** Adrian Gherman, Peter E Chen, Tanya M Teslovich, Pawel Stankiewicz, Marjorie Withers, Carl S Kashuk, Aravinda Chakravarti, James R Lupski, David J Cutler, Nicholas Katsanis

**Affiliations:** 1 McKusick-Nathans Institute of Genetic Medicine, Johns Hopkins University, Baltimore, Maryland, United States of America; 2 Department of Molecular and Human Genetics, Baylor College of Medicine, Houston, Texas, United States of America; 3 Department of Pediatrics, Baylor College of Medicine, Houston, Texas, United States of America; 4 Texas Children's Hospital, Houston, Texas, United States of America; 5 Wilmer Eye Institute, Johns Hopkins University, Baltimore, Maryland, United States of America; Fred Hutchinson Cancer Research Center, United States of America

## Abstract

The modern synthetic view of human evolution proposes that the fixation of novel mutations is driven by the balance among selective advantage, selective disadvantage, and genetic drift. When considering the global architecture of the human genome, the same model can be applied to understanding the rapid acquisition and proliferation of exogenous DNA. To explore the evolutionary forces that might have morphed human genome architecture, we investigated the origin, composition, and functional potential of *numts* (nuclear mitochondrial pseudogenes), partial copies of the mitochondrial genome found abundantly in chromosomal DNA. Our data indicate that these elements are unlikely to be advantageous, since they possess no gross positional, transcriptional, or translational features that might indicate beneficial functionality subsequent to integration. Using sequence analysis and fossil dating, we also show a probable burst of integration of *numts* in the primate lineage that centers on the prosimian–anthropoid split, mimics closely the temporal distribution of *Alu* and processed pseudogene acquisition, and coincides with the major climatic change at the Paleocene–Eocene boundary. We therefore propose a model according to which the gross architecture and repeat distribution of the human genome can be largely accounted for by a population bottleneck early in the anthropoid lineage and subsequent effectively neutral fixation of repetitive DNA, rather than positive selection or unusual insertion pressures.

## Author Summary

Throughout evolutionary history, fragments of the mitochondrial genome, known as *numts* (for nuclear mitochondrial sequences), have been inserted into the nuclear genome. These fragments are distinct from all other classes of repetitive DNA found in nuclear genomes, not least because they are incapable of mediating their own proliferation. Taking advantage of their unique evolutionary properties, we have used *numts* to improve our understanding of the architecture of the human genome with special emphasis on the mechanism of acquisition and retention of repeat sequences, which comprise the bulk of nuclear DNA. We find that *numts* are unlikely to have any evolutionary benefit driving their retention. Moreover, *numts* are not acquired randomly during evolutionary time. Instead, their rate of acquisition spikes dramatically around pronounced population bottlenecks, in a manner reminiscent of other repeat classes. Therefore, we propose that the primary driving force of repeat acquisition in the genome is not selection, but random genetic drift, whose force becomes pronounced during profound reductions of population size. Our findings support the theory of neutral evolution, according to which random genetic drift exerts an influence on the acquisition of DNA changes that far outweighs the power of positive selection.

## Introduction

The present-day human genome arose from the prosimian ancestor through a series of complex chromosomal and local rearrangements. An important feature of our genome, used frequently to understand the adaptive forces that have led to its present-day topology, is the common prevalence of repetitive sequences. Analyses of the *Alu* family, a 300-bp, primate-specific retrotransposon that represents the most abundant class of repeats [1], have indicated that they underwent a seemingly rapid proliferation at two major evolutionary junctions: the prosimian-anthropoid split some 37–55 million years ago (mya) and the platyrrhine/catarrhine split thereafter [2]. Some studies have pointed to a correlation between retrotransposon expansion and speciation [3,4] and have suggested that the unidirectional proliferation of more than ten copies of the retrotransposon [1,5] might provide a useful marker for tracing phylogeny [6,7].

Despite the apparent importance of repeat expansion to understanding the origins of the human genome, the mechanisms of repeat proliferation are poorly understood. For *Alu* repeats, a model of increased retrotransposition activity has been proposed [8], but the underlying evolutionary forces behind such a mechanism are unclear.

To investigate the evolutionary forces that might govern the acquisition and retention of repetitive elements in the human genome, we selected an entirely different class of repeat whose mechanisms for insertion, deletion, and selection are so fundamentally different from *Alu* that any commonality in their evolutionary dynamic is probably due to the fact that they share the same population size, rather than any underlying biological mechanism.

We focused on *numts* (nuclear mitochondrial sequences/pseudogenes), partial copies of the mitochondrial genome found abundantly in chromosomal DNA. Since the first demonstration of organellar sequence embedded in nuclear DNA [9], *numts* have been described in several mammalian species, as well as over 70 other eukaryotes [10–12]. The varying level of homology between these sequences and the present-day mitochondrial genome, as well as population and family polymorphisms, indicates that the nuclear transfer of mtDNA is an ongoing process [13–21,28]. In contrast to plants and fungi, in which *numts* have arisen from both RNA- and DNA-mediated mitochondrial DNA (mt-DNA) transfers [22], the origin of *numts* in metazoans has been proposed to be DNA- rather than RNA-mediated [23–25]. As such, the *numts* family of repeats represents a useful tool for evolutionary analysis since its proliferation mechanism is distinct from *Alu* elements, in that it does not rely on retrotransposition.

## Results

### An Updated *numts* Map of the Human Genome

**Figure 1:**
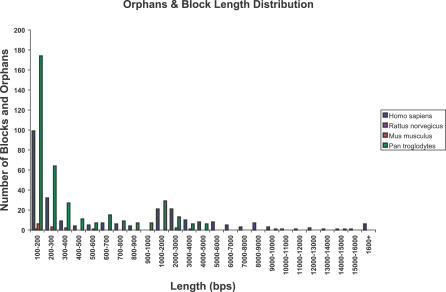
Distribution of *numts* and Fragment Length in the Human, Chimp, Mouse, and Rat Genomes

We first used the assembled human genomic sequence (Build 36) to investigate the prevalence and distribution of *numts* in the human genome. Using default sequence alignment selection criteria (*e*-value <10), we identified 2,329 *numts* fragments that range in size from <100 bp to 16 kb (Figure 1), a number consistent with previous studies [19,23,26]. Fine-mapping of *numts* showed many instances in which multiple, seemingly independent, fragments map in close proximity to one another, suggesting a higher-order organization*,* whereby each *numts* does not represent an independent integration, but is rather a fossil of a single ancestral integration (Table S1). Clustering of such *numts* blocks indicated that the human genome likely contains in excess of ∼1,200 *numts* elements (Table S2). A similar analysis of the mouse and rat assembled genomes showed a marked *numts* paucity, with 636 and 529 *numts* fragments, respectively. By contrast, the recent draft of the chimp genome contains numbers comparable to humans, ≥1,280 *numts*, suggesting that these elements might have undergone a dramatic expansion in the primate lineage (Table S2). These observations are unlikely to be due to inappropriate exclusion of *numts* sequences from the draft genome assemblies, since analysis of the raw trace data (i.e., all individual preassembly sequence reads) showed a similar percent identity distribution of putative *numts,* with both sequence collections peaking at 82%–88% identity with the present-day mitochondrial sequence (data not shown).

### Verification of the *numts* Complement of the Human Genome

**Figure 2:**
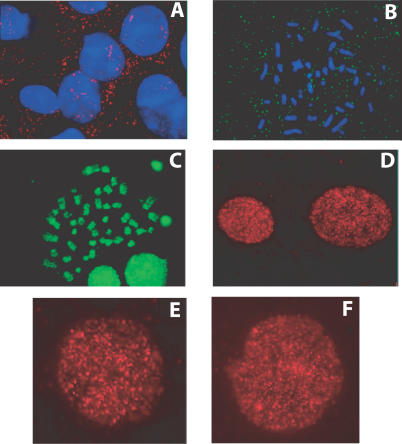
Visualization of *numts* in Cultured Cells (A) Human interphase nuclei after FISH with complete mt-DNA as a probe. Note that the vast majority of the probe hybridized to the mt-DNA in remaining cytoplasm. (B) A similar pattern was observed on a metaphase chromosome spread. (C) The mt-DNA-free metaphase and interphase chromosomes yielded “painting” characteristics when hybridized with mt-DNA. (D–F) The interphase nuclei of chimpanzee (D), gorilla (E), and orangutan (F) after depletion of mt-DNA and hybridization with human mt-DNA probe.

Prior to further analysis, we corroborated our computational data in two ways. First, we performed flourescent in situ hybridization (FISH) with mtDNA as a molecular probe on interphase and metaphase nuclei of mtDNA-depleted cells as target DNA. Consistent with the predicted abundance of *numt* in the nuclear genome, we detected fluorescence signals scattered along each chromosome (Figure 2). We observed a similar pattern on chromosomes of mtDNA-depleted lymphoblast cells from chimp, gorilla, and orangutan (Figure 2). These data indicate that the *numts* element is distributed widely in the genomes of these species and that the actual *numts* population is probably larger than our computational predictions, potentially reflecting our criteria for *numts* identification. In addition, amplification from a monochromosomal hybrid panel and subsequent sequencing of 24 randomly selected nucleo–*numts* junctions, showed that in each case the amplification and sequence data matched exactly with the computationally predicted sequence of each *numts* (data not shown).

### *Numts* Proliferation Is Unlikely to Be Sequence Context Dependent

We next investigated *numts* proliferation. Previous studies have indicated that the mechanism of integration of these repeat elements into the genome is distinct from retroviral insertion or recombination [10], thus enabling us to study the acquisition characteristics of exogenous DNA in a genome context-independent fashion. To identify a subpopulation of *numts* that arose by independent integrations, rather than a single integration followed by subsequent segmental duplication, we first correlated the positions of all identified *numts* with the segmental duplication map. In agreement with previous studies founded on *numts* base substitution rates [13], we determined that although some *numts* proliferated through chromosomal rearrangements, the majority of *numts* acquisition of the genome reflects independent integration; some 3%–5% of build 36 has been identified as segmental duplication [27], and only 4% of all *numts* map to these regions. To further confirm these observations, we compared 500 bp of nuclear sequence on either side of each putative integration and found no similarities among the nuclear junction sequences (data not shown).

**Figure 3:**
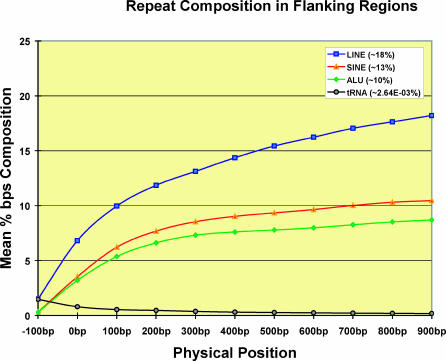
The Insertion of *numts* Is Repeat-Independent Plot comparing the average repeat composition of the nucleo–*numts* junctions of 266 independent *numts* with 50,000 random sequence fragments of equivalent length. The *x*-axis shows the distance from the estimated end of the *numts* (position zero), i.e., the region over which *e* < 10, the corresponding average repeat content of the human genome is shown in the legend box in parentheses. The *y*-axis depicts the percentage composition of various repeat classes (given in the box); all repeat classes are under or at genome-wide density within 500 bp of the *numts* junction, indicating no major repeat involvement in the integration preference of *numts*.

We next asked whether *numts* integration is likely to be genome sequence independent by evaluating the sequence characteristics of nucleo–*numts* junctions. First, we asked whether there is any observable enrichment for a recognizable element at repeat junctions. A comparison of 1 kb of flanking nuclear junction sequence surrounding 266 *numts* with the entire human genome showed an initial deficit of repeats, returning to genome-wide levels 500–600 bp past the insertion boundary (Figures 3 and S1). This suggested that: (a) there is no repeat excess at the boundary and (b) the true boundary probably lies 500–600 bp away from our initial prediction. In addition, the possibility of a TE (transposable element) insertional mechanism was also deemed unlikely, since we found no evidence of sequence duplication anywhere within the 1kb region that flanks the boundaries of each *numt*.

Our data suggest that the human genome has probably acquired a minimum of several hundred *numts*, most of which arose in an ancestor as independent events, in a process that is still ongoing [28] and can have detrimental effects to gene function [29]. Even though the mechanism of insertion of *numts* is clearly different from that of *Alu* elements, especially since *numts* cannot mediate their own proliferation, similarities or differences in the fitness consequences of those insertions are less obvious. Although *numts* are unlikely targets for unequal exchange events, they might contain potentially functional genes that could be co-opted into some nuclear role. Thus, we assessed for possible fitness effects of *numts* insertion by examining their positional preference in the genome, as well as their transcriptional and translational potential.

### *Numts* Are Unlikely to Have Been Often Co-opted for Transcription Control or Translation

**Figure 4:**
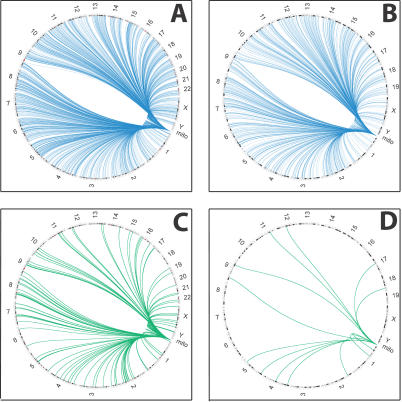
*numts* Distribution across Homo sapiens and Mus musculus Genomes (A) and (B) are illustrating the *numts* distribution across the entire Homo sapiens (A) and Mus musculus (B) genome. The blocks are also represented in (C) for Homo sapiens and (D) for *Mus musculus,* respectively.

To interrogate whether *numts* have positional preference, we determined the relative distribution of all large *numts* arisen by independent integrations with respect to the coding sequence distribution of the genome. We conducted two tests, one for *numts* >1 kb (*n* = 99) and one for *numts* >500 bp (*n* = 121). None of the *numts* considered for the two experiments occurred in exons. In build 36, the fraction of the intronic human genome is ∼28.85%. The percentage of intronic *numts* is 22.3% (22/99; binomially *p* = 0.086) for *numts* >1 kb and 21.5% (26/121; *p* = 0.042) for the those >500 bp. Thus, *numts* appear to be distributed relatively randomly in the genome (Figure 4), but a slight statistical tendency towards intergenic intervals was observed, probably underlying the higher potential of intragenic insertions for a deleterious effect. Overall, we conclude that *numts* position within the genome provides little evidence of its use for transcriptional control.

Next, we considered the possibility that *numts* might have functionality at the mRNA level. We first examined whether *numts* are transcribed, by interrogating each *numts* against dbEST. To reduce the incidence of matches with dbEST due to short segments of sequence, we restricted our queries to *numts* with length greater than 1 kb and *numts* longer than 500 bp. Of the 99 *numts* >1 kb evaluated, (23/99) 23.23% were represented in dbEST, also from the 121 *numts* >500 bp considered, (33/121) 27.27% were found in dbEST. Reverse-transcriptase PCR (RT-PCR) of 24 randomly selected, nonoverlapping ESTs also indicated that the majority of these sequences represent *bona fide* transcription, since in 22 instances we amplified successfully the correct fragment from a panel of eight adult human RNA samples by RT-PCR (data not shown). However, we found no positional preference for putatively transcribed *numts,* suggesting that *numts* mRNA is unlikely to exert a *cis*-acting regulatory role.

**Figure 5:**
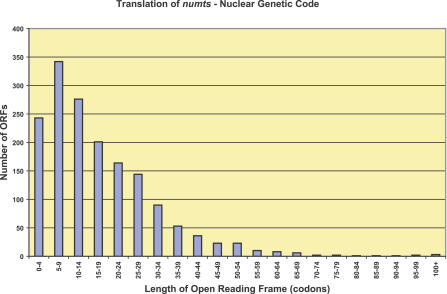
*Numts* Sequences Have Little Overt Translational Potential Each *numts* was translated using the nuclear genetic codes, and ORF lengths were plotted. The *x*-axis depicts ORF length in bins of five codons. The *y*-axis shows the number of ORFs with lengths within each range. The mean ORF lengths for the nuclear and mitochondrial translations are 19 codons and 17 codons, respectively.

Finally, we considered the possibility that the introduction of *numts* into the genome provided the template for new protein sequence, despite the fact that the nuclear and mitochondrial genome have different genetic codes. We therefore examined the translational potential of each *numts* in all six reading frames (Figure 5). Translating with the nuclear code results in a distribution of open reading frame (ORF) lengths indistinguishable from random sequence (3/64 codons are stop, therefore random sequence will generate ORF sizes with a mean size of ∼20 codons). Although there is a slight excess of long ORFs (suggesting that a small fraction of *numts* might be translated), the overall distribution of ORF lengths is approximately exponential with a mean length of 5–15 codons.

Cumulatively, our data suggest that there is little evidence for overt functionality for the majority of *numts,* and although we cannot formally exclude the possibility that some individual repeats have a biological role (and may thus be obvious targets for positive selection), the overall population of this repeat is likely to be on average evolutionarily neutral or deleterious.

### Accumulation of *numts* in a Temporal Burst

**Figure 6:**
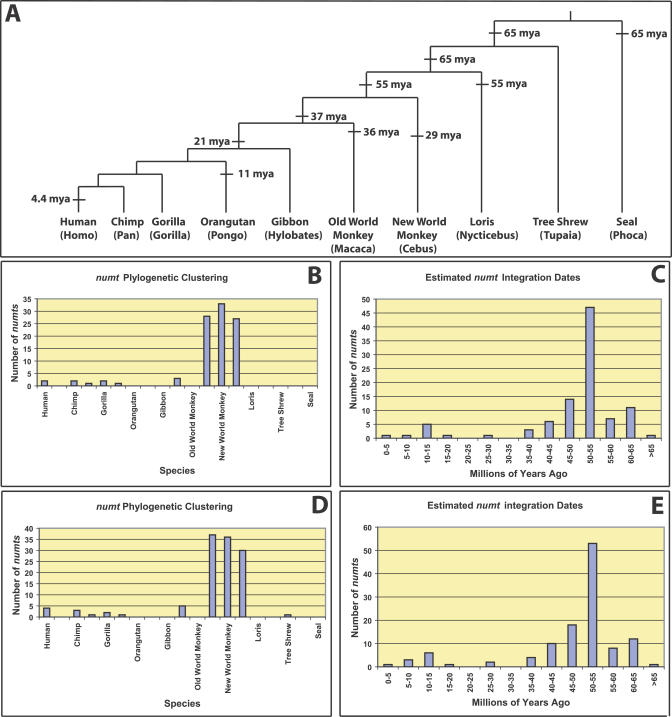
Burst of *numts* Fixations (A–E) Phylogenetic tree of primates and outgroup (seal) used in alignment of *numts* and mitochondrial sequences. Calibration points used in estimation of *numts* age are shown in units of mya and are derived from fossil dating evidence [30]. Histogram of *numts* position within phylogenetic trees was inferred for *numts* with length greater than 1kb (B) and *numts* longer than 500 bp (D). Each *numts* was aligned to the mitochondrial sequences of the species shown, and phylogenetic trees were inferred using a neighbor-joining algorithm. Any *numts* that was grouped in a sub-tree with one of the extant mitochondrial sequences has been depicted in the bin labeled with that species name. Any *numts* that formed its own branch between two species has been depicted in the bin between those labeled with the names of the two species. Histograms of estimated dates of *numts* integrations were also obtained for *numts* with length greater than 1 kb (C) and *numts* longer than 500 bp (E). Each inferred tree was analyzed with the program dating using the fossil calibration dates shown in (A). The *x*-axis depicts the estimated date of integration in mya. The *y*-axis shows the number of *numts* with estimated dates in each range.

To gain a better understanding of the evolutionary dynamics of *numts,* we sought to determine the most likely time of integration of each *numts* into the nuclear genome. To do so, we aligned each *numts* to a collection of complete modern mtDNA sequences spanning the primate radiation. The time of each integration was inferred independently with multiple fossil calibration points [30] under an overdispersed model of molecular evolution, accounting for variation in evolutionary rates within and between *numts* and the extant mitochondria (Figure 6A) [31]. In contrast to an expectation of progressive *numts* accumulation during evolutionary time, we were surprised to find an apparent burst of *numts* integrations at approximately 54 mya. Focusing first on *numts* >1 kb in length, we found that ∼76% out of the 99 unique integration events, have an estimated time of insertion within 10 mya of 54 mya (Figure 6C). Next, we considered the *numts* >500 bp, and from 121 unique integration events ∼75% also occurred within 10 mya of 54 mya (Figure 6E). Thus, 75%–80% of all *numts* integrations appear to have occurred within a relatively narrow window of time around 54 mya, between the New World Monkey and Old World monkey transition (Figure 6B and 6D). Importantly, this estimate is likely to remain true irrespective of assumptions regarding the nucleotide substitution rate of *numts* versus mtDNA, as judged by a confidence interval plot of the 121 500-bp+ *numts* (Figure S2).

## Discussion

Most *numts* appear to have accumulated in a 10-millon-year window centered around 54 mya. Importantly, other repetitive elements show a similar pattern, including *Alu* repeats [2,32] and processed pseudogenes [33], suggesting a period of intense DNA acquisition in the ancestral genome. Given that *numts* are markedly distinct from *Alu* repeats and other retrotransposons in both their mechanism of integration, as well as proliferation (especially since *numts* lack the ability to self propagate), the force behind the expansion of repeats is likely independent of genome structure. This notion is further supported by the fact that the boundaries of *numts* integration show no marked enrichment for any sequence elements (Figure 3). It will always remain a formal possibility that *numts* integration was primarily driven by positive selection for the accumulation of these elements. However, the absence of overt functionality of *numts* in the present-day genome, and the fact that *numts* integration is a continuing process [10], principally detected because of its disease phenotype, argues against this hypothesis. Thus, we arrive at three important questions concerning the evolutionary history of *numts*: (1) Why did so many *numts* accumulate approximately 54 mya? (2) Why did they stop accumulating? (3) Why does this time period correspond temporally with accumulation of other entirely unrelated genetic elements?

The theory that governs the evolutionary dynamics of TEs can provide important clues about the mechanism of acquisition and retention of *numt, Alu,* and other repeat elements in the human genome. In an infinite sized population, the change in the mean number of TEs per individual, 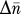 , is approximately

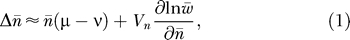
where *V_n_* is the variance in copy number between individuals, μ is the rate of new insertions, ν is the rate of new deletions, and 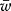 is population mean fitness [34,35]. Thus, in an infinite-sized population, TE copy number is governed by a balance between the effects of new insertion, new deletion, and selection. By contrast, in a finite population, Equation 1 will approximately hold whenever 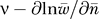 is much bigger than 1/*N,* where *N* is the effective size of the population. If 1/*N* > 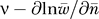 , TE copy number will rise (if the insertion rate is greater than the deletion rate) or fall (if deletion is more frequent than insertion). Thus, a sudden change to TE copy number could reflect a sudden decrease in population size, shifting the balance between selection and mutation forces to one where genetic drift ruled and allowed for unbounded increase in TEs. The Liu et al. hypothesis [8], on the other hand, suggests that the increase in *Alu* copy number may have resulted from a sudden increase in μ, the rate of insertion.

If we assume that *numts* integrations are principally weakly deleterious on average (a notion supported by their ongoing contribution to disease), an examination of Equation 1 suggests that a simple population size hypothesis can provide an answer to all three of our questions. We begin by assuming that prior to 54 mya, the effective population size of the primate ancestor was relatively large, leading to an insertion/deletion/selection equilibrium with *numts* count being few and held stable at that low value (which is consistent with the relative paucity of *numts* in the mouse and rat lineages). However, if we further assume that at approximately 54 mya, effective population sizes declined dramatically, to a point where 1/*N* > 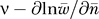 , then *numts* would for evolutionary purposes become effectively neutral, and, during their period of effective neutrality, they would accumulate with little selective check, at a rate proportional to μ−ν (the difference between the insertion and deletion rates of an element). Since population size changes affect everything in the genome, elements with high insertion rates (such as *Alu* elements) would be expected to accumulate in great abundance (which they do), whereas elements with relatively low insertion rates (such as *numts*) also accumulated, albeit in fewer numbers. Finally, a subsequent increase in effective population size would shift the population back into an insertion/deletion/selection equilibrium, and the period of accumulation would end.

Clearly, the assumptions of relative *numts* neutrality and of a population bottleneck at ∼54 mya cannot be proven definitively. Nonetheless, based on observations of the landscape of the present day genome of humans and other species, our proposed evolutionary model has many attractive features. First, it provides a common mechanism (decline in effective population size) for the increase in numbers of unrelated repetitive elements. Second, it explains both the sudden increase in repetitive DNA, and the later cessation of the increase. Third, the timing of the event, occurring immediately prior to the adaptive radiation of monkeys, is highly evocative, reminiscent of a Wrightian/Simpsonian view of speciation: a large population of stem anthropoids splintered into multiple demes. One or more such small deme accumulated repetitive DNA in abundance, which in turn may have served as a post-zygotic reproduction barrier with the original population. This isolated deme ultimately speciated and underwent an adaptive radiation into the anthropoid primates. It is notable (and unlikely to be coincidental) that the timing of the repeat-inferred bottleneck at ∼54 mya coincides with a major environmental disturbance at the Paleocene–Eocene boundary (∼55 mya), which strongly effected global mammalian faunas and corresponds to the first appearance of primates in the fossil record of the northern hemisphere [36].

This hypothesis suggests that human and primate genomic architecture, with its abundance of repetitive elements, arose primarily by evolutionary happenstance; although it remains plausible (and indeed, probable) that some integrons were subsequently co-opted into an interesting use such as X inactivation [37] or perhaps gene regulation [38], these complicated hypotheses do not explain satisfactorily the bulk of human genomic architecture. A simple explanation states that the population that gave rise to primates was quite small, and as a result the genomic architecture of primates may have resulted from effectively neutral integrations of repetitive DNA.

## Materials and Methods

### Data collection.

Human mitochondrial genome sequence was compared against genomic sequence with BLAST (NCBI Build 36). The process was repeated for the mitochondrial sequence of chimp, mouse and rat against the following draft builds: chimp Build 2 (October 2005), mouse Build 33 (May 2004; mm5), and rat Version 3.1 (June 2003; rn3). In each case, hits that scored with an expected value <10 were retained. All annotations (repeat classes, gene boundaries, etc.) were taken from the University of California Santa Cruz genome browser, http://genome.ucsc.edu/.

### Block assignment.

Blast hits were sorted by genomic position, and the differences (“gaps”) between consecutive hits on both the genomic and mitochondrial scales were calculated. Pairs of hits that had a ratio of mitochondrial gap size to genomic gap size between 0.9 and 1.1 were assigned to be in the same block (hand picked). The *numts* distribution plots were created using Circos (http://mkweb.bcgsc.ca/circos/).

### Preparation of mt-DNA as a molecular probe for FISH.

We used high-molecular-weight genomic DNA and highly purified mt-DNA from HeLa cells (kindly provided by Samuel E. Bennett, Oregon State University, Corvallis, Oregon, United States) for PCR. For generating molecular probes in FISH experiments, we used two different PCR products: the complete mitochondrial genome (16.3 kb) amplified with the TaKaRa PCR kit (Fisher Scientific, https://new.fishersci.com/), using conditions as described [39]. Alternatively, we designed seventeen PCR primer sets and amplified overlapping ∼1-kb fragments, covering the entire mt-DNA sequence. Primers and detailed PCR conditions are available upon request.

### Primate cell lines.

The nonhuman primate immortalized Epstein–Barr virus–stimulated cell lines of common chimpanzee *(Pan troglodytes),* lowland gorilla (*Gorilla gorilla,* CRL 1854), and orangutan *(Pongo pygmaeus),* were purchased from the American Type Culture Collection (ATCC, http://www.atcc.org/). The pygmy chimp *(Pan paniscus)* lymphoblast sample was kindly provided by D. Nelson at Baylor College of Medicine, Houston, Texas, United States.

### Isolation of human and primate cell lines depleted of mitochondrial DNA.

Human and primate lymphoblasts were depleted of mt-DNA according to the slightly modified protocol of King and Attardi [40]. Cells were grown for 5–6 d in DMEM enriched with 10% FCS glucose (4,500 mg/ml), sodium pyruvate (1 mM), uridine (50 μl/ml), and ethidium bromide (50 μl/ml).

### Fluorescence in situ hybridization.

Normal and mt-DNA-depleted lymphoblasts were harvested using standard methods. FISH was performed on metaphase and interphase cells as described [41]. Briefly, PCR products were labeled with biotin (Life Technologies-GibcoBRL, http://www.invitrogen.com/) or digoxigenin (Boehringer Mannheim, http://www.roche.com/) by nick translation. Biotin was detected with FITC-avidin DCS (fluoresces green; Vector Labs, http://www.vectorlabs.com/) and digoxigenin was detected with rhodamine-anti-digoxigenin antibodies (fluoresces red; Sigma, http://www.sigmaaldrich.com/). Chromosomes were counterstained with DAPI diluted in Vectashield antifade (Vector Labs). Cells were viewed under a Zeiss Axioskop fluorescence microscope (http://www.zeiss.com/) equipped with appropriate filter combinations. Monochromatic images were captured and pseudocolored using MacProbe 4.2.2/Power Macintosh G4 system (Apple, http://www.apple.com/; Perceptive Scientific Instruments, http://www.perceptive.co.uk/).

### Repeat composition analysis of block flanking sequences.

The flanking sequence composition of 266 *numts* was compared to 50,000 randomly chosen sequences drawn uniformly from the human genome. For each flanking sequence, and each randomly drawn sequence, the proportion of the sequence covered by various repeat families (*Alu*, L1, MALR, etc.) and repeat classes (SINE, LINE, LTR, etc.) was calculated and the repeat composition of each category was evaluated with a *t*-test.

### Amplification of *numts* junction fragments.

Once the composition and distribution of *numts* blocks was established, we designed primers to amplify 250–400-bp junction fragments whereby one primer was anchored at unique nuclear sequence and the other primer was situated at the edge of a *numts* block. We performed PCR using standard condition on human–rodent monochromosomal hybrids as described [42].

### Expression analysis of ESTs.

We designed primers from ESTs that matched human *numts* with >98% identity over 200 bp of sequence. To ascertain their expression patterns, we generated amplicons from eight adult human cDNAs (Clontech, http://www.clontech.com/) according to manufacturer's instructions.

### Translational potential.

Each *numts* was translated in all six possible reading frames. An ORF was defined as the sequence between two stop codons, and the frame with the longest mean ORF length was chosen for inclusion in the analysis. *Numts* were translated using the nuclear genetic codes (stop codons TAA/TAG/TGA).

### Estimation of the time of integration events.

Each *numts* was aligned individually with ClustalW (http://www.ebi.ac.uk/clustalw/) to a collection of complete modern mtDNA sequences spanning the primate radiation, rooted by a carnivore outgroup. All pairwise per-site divergences were calculated with the PHYLIP program (http://evolution.genetics.washington.edu/phylip.html) dnadist, using a Kimura 2-parameter substitution model to correct for multiple hits. For each *numt,* the evolutionary tree was inferred by both parsimony (using the PHYLIP dnapars program) and neighbor-joining (using the PHYLIP program neighbor). In all cases the expected phylogeny [2] of the primate and outgroup was recovered, but the exact position of the *numts* varied slightly (see below). Once the tree was inferred for each *numt,* the number of substitutions per branch was estimated by least-squares minimization using the PHYLIP program fitch with default parameters.

To account for any potential uncertainty in the divergence time between extant primates, nonconstancy of evolutionary rates within and among different functional portions of the extant mtDNA, and perhaps vastly different rates of evolution among nuclear pseudogene copies of mtDNA and extant functional mtDNA, the time of each integration was inferred with dating [31], under a stationary substitution model with multiple fossil calibration points [30]. In all cases, the stationary model fit better than the constant rate Poisson model by several orders of magnitude. Confidence intervals for each integration were also calculated [31].

## Supporting Information

Figure S1 Expanded Version of Figure 3 to Show the Detailed Distribution of All Repeat Classes at the Nucleo–*numts* Boundary (271 KB PDF)

Figure S2 Point Estimates and Confidence Intervals for the 121 *numts* Insertions >500 bp Although some of the confidence intervals are asymmetric, probably reflecting a different rate of evolution between *numts* chromosomal DNA and mtDNA, most confidence intervals include the 45–55 mya range.
(121 KB PDF)

Table S1 Orthologous Gap Sizes between Genome and Mitochondrial Genome (20 KB PDF)

Table S2 Summary of *numts* Sequences in the Human, Chimp, Mouse, and Rat Genomes (29 KB PDF)

### Accession Numbers

The National Center for Biotechnology Information (NCBI) Genbank (http://www.ncbi.nlm.nih.gov/sites/entrez?db=Nucleotide) accession number for the human mitochondrial genome sequence discussed in this paper is NC_001807.

## Abbreviations

mt-DNA: mitochondrial DNA
FISH: flourescent in situ hybridization
*numts,*: million years ago
*numts,*: nuclear mitochondrial sequence
ORF: open reading frame
RT-PCR: reverse-transcriptase PCR
TE: transposable element
