# Supplementary material for: Population Bottlenecks as a Potential Major Shaping Force of Human Genome Architecture
Source: PLoS Genet. 2007 Jul 20;3(7):e119. doi: 10.1371/journal.pgen.0030119 (PMC1925129; doi:10.1371/journal.pgen.0030119)
Supplement: Supplementary file 1 [file pgen.0030119.sg001.pdf]

Suppl. Fig. 1

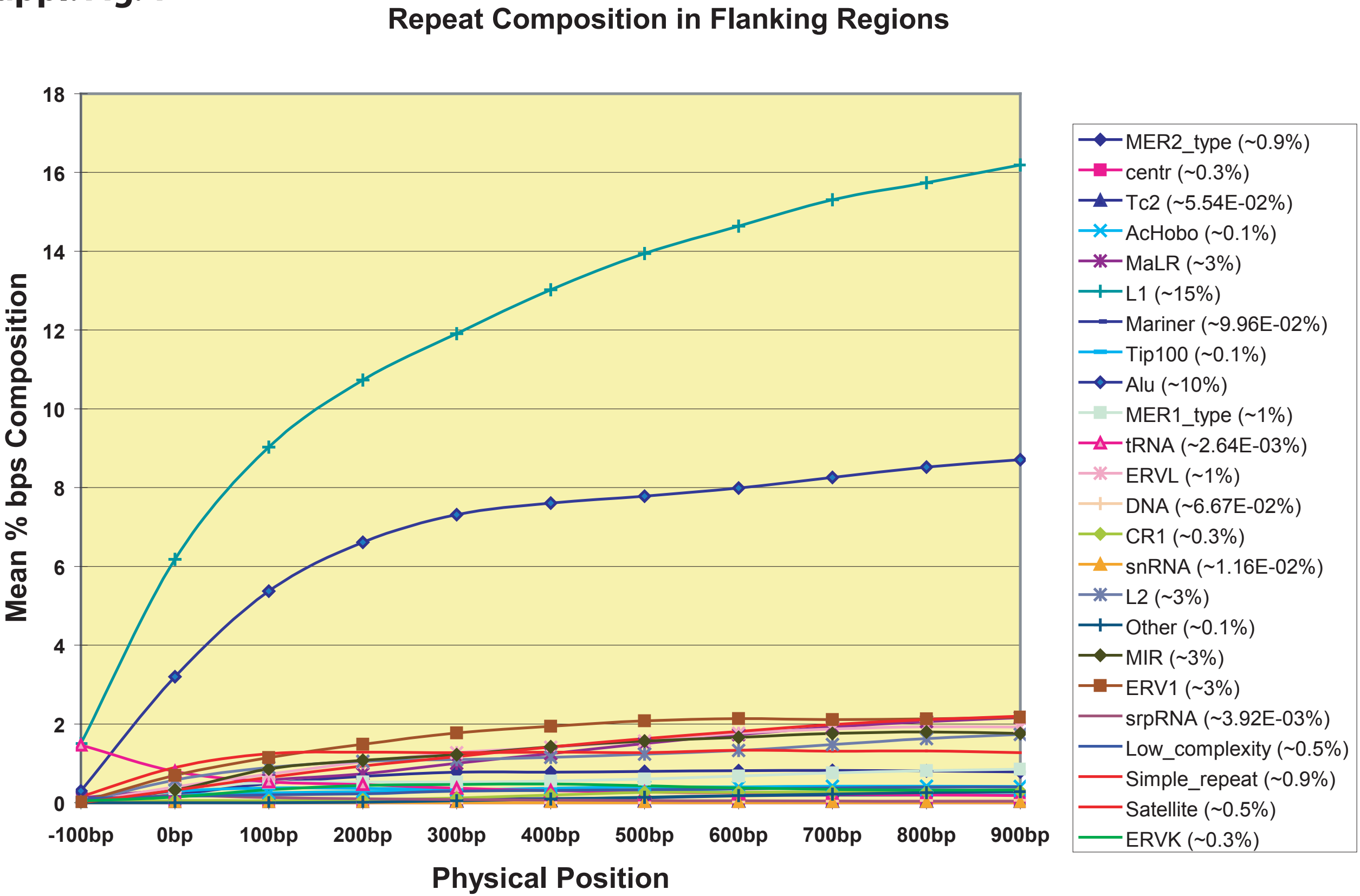

Repeat Composition in Flanking Regions
Mean % bps Composition
18
16
14
12
10
8
6
4
2
0
-100bp
0bp
100bp
200bp
300bp
400bp
500bp
600bp
700bp
800bp
900bp
Physical Position
MER2_type (~0.9%)
centr (~0.3%)
Tc2 (~5.54E-02%)
AcHobo (~0.1%)
MaLR (~3%)
L1 (~15%)
Mariner (~9.96E-02%)
Tip100 (~0.1%)
Alu (~10%)
MER1_type (~1%)
tRNA (~2.64E-03%)
ERVL (~1%)
DNA (~6.67E-02%)
CR1 (~0.3%)
snRNA (~1.16E-02%)
L2 (~3%)
Other (~0.1%)
MIR (~3%)
ERV1 (~3%)
srpRNA (~3.92E-03%)
Low_complexity (~0.5%)
Simple_repeat (~0.9%)
Satellite (~0.5%)
ERVK (~0.3%)
