# Supplementary material for: Population Bottlenecks as a Potential Major Shaping Force of Human Genome Architecture
Source: PLoS Genet. 2007 Jul 20;3(7):e119. doi: 10.1371/journal.pgen.0030119 (PMC1925129; doi:10.1371/journal.pgen.0030119)
Supplement: Supplementary file 2 [file pgen.0030119.sg002.pdf]

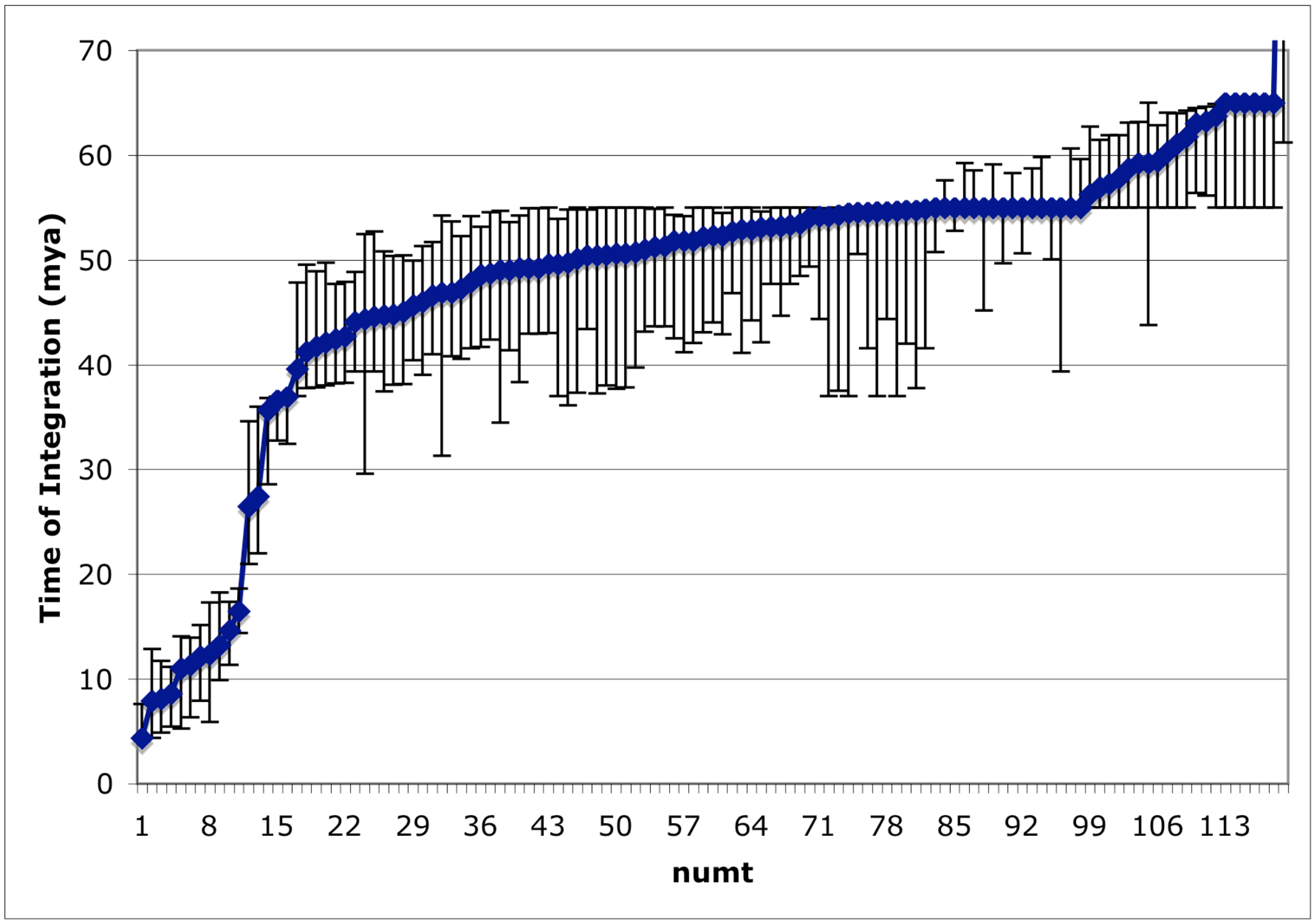

70
60
50
40
30
20
10
0
Time of Integration (mya)
1
8
15
22
29
36
43
50
57
64
71
78
85
92
99
106
113
numt

Suppl. Fig. 2
