# Supplementary material for: Population Bottlenecks as a Potential Major Shaping Force of Human Genome Architecture
Source: PLoS Genet. 2007 Jul 20;3(7):e119. doi: 10.1371/journal.pgen.0030119 (PMC1925129; doi:10.1371/journal.pgen.0030119)
Supplement: Supplementary file 3 [file pgen.0030119.st002.doc]

Suppl. Table 2. Summary of numt sequences in the human, chimp, mouse and rat genomes.

|  | Fragments | Blocks | Orphans | Duplicons | Integrons |
| --- | --- | --- | --- | --- | --- |
| Homo sapiens | 2329 | 112 | 1060 | 95 | 1076 |
| Pan troglodytes | 1728 | 196 | 1084 | N/A* | N/A* |
| Mus musculus | 636 | 14 | 600 | 11 | 603 |
| Rattus norvegicus | 529 | 2 | 527 | 7 | 522 |

Fragment : local alignment with an expected value <10

Block: a set of two or more fragments that represent a single ancestral integration

Orphan: fragment not belonging to any observed blocks

Duplicon: fragment or block that resides within a segmental duplication

Integron: fragment or block that does not reside within any segmental duplication

*segmental duplication for Pan troglodytes not yet available
